# Excitation of the Auditory System as a Result of Non-invasive Extra-Cochlear Stimulation in Normal Subjects and Tinnitus Patients

**DOI:** 10.3389/fnins.2018.00146

**Published:** 2018-03-23

**Authors:** Marzena Mielczarek, Arnaud Norena, Winfried Schlee, Jurek Olszewski

**Affiliations:** ^1^Department of Otolaryngology, Laryngological Oncology, Audiology and Phoniatrics, Medical University of Lodz, Lodz, Poland; ^2^Laboratoire Neurosciences Intégratives et Adaptatives, Aix-Marseille Université, Marseille, France; ^3^Department for Psychiatry and Psychotherapy, University of Regensburg, Regensburg, Germany

**Keywords:** tinnitus, electric stimulation, cathodal stimulation, anodal stimulation, auditory percept, ear, sensorineural hearing loss

## Abstract

One of possible approach that may suppress tinnitus is electrical stimulation of the ear. At first invasive techniques were used (promontory or round window stimulation), nowadays a non-invasive method, namely hydrotransmissive electric stimulation (ES) through external acoustic canal, has been developed. The aim of the study is to investigate the effect of applying ES with positive and negative current polarities on the ears of healthy subjects and on the tinnitus ears of patients with tinnitus. This comparison further clarifies the mechanisms of operation of non-invasive extra-cochlear ear ES. A second aim is to assess the effects of ES on tinnitus in tinnitus patients. The material was composed of two groups: tinnitus group—49 patients suffering from tinnitus, and healthy students group—34 healthy individuals. ES was performed with the use of a custom-made apparatus. The active, silver probe–was immersed inside saline filling external ear canal. The passive electrode was placed on the forehead. Positive and next negative DC stimulation was provided with the use following frequencies: 0.25, 1, 2, 3, 4, 5, 6, 7, 8 kHz. We checked for the presence of the auditory percept (AP) and, if AP was present, the minimum current amplitude necessary to produce AP was measured. In our research both positive and negative polarities were efficient to evoke AP in the participants. This effect, however, was more pronounced for positive polarity in no tinnitus and normal hearing individuals (healthy students group). In the tinnitus group, current intensity needed to evoke AP was higher than in the healthy students group. However, comparing normal hearing vs. hearing loss patients within the tinnitus group, we did not observe the relationship between hearing threshold and current intensity evoking AP. Afterwards, we analyzed the effect of multi-frequency ES on tinnitus. It appeared to be effective in 75% of tinnitus ears (with a high score of disappearance–22%). Our study proved that extracochlear ES with positive and negative current was efficient to stimulate the auditory system. Stimulating tinnitus ears with two polarities we obtained a higher ratio of improvement (75%) comparing to positive stimulations.

## Introduction

Tinnitus, an auditory percept that is not induced by any acoustic stimulation in the environment, is largely prevalent in the general population and can dramatically impair the quality of life. Tinnitus can be classified as peripheral or central: Peripheral tinnitus is defined as resulting from aberrant neural activity in the cochlear nerve propagating all the way up to the auditory centers, while central tinnitus is defined as resulting from aberrant neural activity generated in the auditory centers, when cochlear spontaneous activity is reduced or absent (in the case of severe hearing loss) (Noreña, 2015). However, according to some models of tinnitus, it is possible that tinnitus may have a mixed origin, i.e., peripheral and central (Noreña, 2011, 2015). It has been proposed that this mixed tinnitus can result from an amplification of residual cochlear nerve activity (Mulders and Robertson, 2009; Noreña, 2011). Various approaches to treatment can also be used. Central tinnitus can be treated by interfering with the tinnitus-related central mechanisms (Noreña and Eggermont, 2005; Pantev et al., 2012; Tass and Popovych, 2012); these approaches assume that tinnitus results from the central changes after hearing loss, and that these changes can be reversed by appropriate stimulation. Alternatively, peripheral or mixed tinnitus is treated by suppressing or reducing the spontaneous activity in the cochlea; more precisely, if tinnitus is caused by hyperactivity in the cochlear nerve, then a clinical approach should reduce this activity. This is the rationale for pharmacological treatment using NMDA antagonists (Puel, 2007; Ruel et al., 2008; van de Heyning et al., 2014).

Another approach that may suppress tinnitus-related cochlear activity is electrical stimulation (ES), first used when House reported total tinnitus suppression after cochlear implantation (House, 1976). Invasive techniques have been found to remove tinnitus in 43–60% (Aran and Cazals -transtympanal stimulations of the promontorium and round window respectively), 45% (Rubinstein–round window stimulation) in 69–77% (Ito and Sakakihara–promontory test and cochlear implant respectively) of cases depending on the study (Cazals et al., 1978; Portmann et al., 1979; Ito and Sakakihara, 1994; Rubinstein et al., 2003). Further non- invasive techniques have been developed, with success rates of 62.2% (Lee et al.—transcutaneous ES of the auricle), 50% (Kuk et al.—eardrum) and 40.4% (Maini and Deoganonkar – stimulation of the mastoid) (Portmann et al., 1979; Chouard et al., 1981; Shulman, 1987; Quaranta et al., 2004; Mielczarek and Olszewski, 2014). Nowadays electrical stimulation is used as a test to predict post-operative profits before cochlear implantation. During non-invasive extratympanic ear stimulation (via a ball-shaped electrode dipped in saline in external ear canal) sound perception is considered an evidence of acoustic nerve excitation, also confirming restored function (Bochenek et al., 1989; Skarzynski et al., 1999; Dehmel et al., 2008).

The first experiments with ES at the Medical University of Lodz date back to the 1980s. At first, invasive transtympanic promontory positive DC stimulations were used with a success rate reaching 42% (Konopka et al., 2001). Later, a non-invasive approach was used: hydrotransmissive stimulations through the external acoustic canal with the use of positive DC. Improvement was obtained directly after treatment in 37.8% of cases; however, the follow up after 1 month found the success ratio had increased to 51.3%. A comparison with a placebo group showed statistically significant differences indicating the value of the method (Mielczarek and Olszewski, 2014).

Although many studies have reported tinnitus improvement or tinnitus suppression after ES (Shulman, 1987; Bochenek et al., 1989; Skarzynski et al., 1999), the exact mechanism of this phenomenon remains unclear. It has been suggested that ES works by increasing the transmission of neurotransmitters in the synapses (Latkowski, 1981), modifying the electrical potentials of the hearing organ (Portmann et al., 1979), or by improving the blood flow in the inner ear and synchronizing the spontaneous impulses in the auditory nerve fibers (Watanabe et al., 1997). Another suggested mechanism of action is by stimulation of the C_2_ dorsal root. Dehmel et al note that the C_2_ fibers, supplying the skin of the retroauricular region and mucosal lining of the tympanic cavity, target cells in the dorsal column nuclei, which then send axons to the dorsal cochlear nuclei (Dehmel et al., 2008). This mechanism may account for the relief of tinnitus experienced after stimulation of the cochlea surface (Møller, 2016).

Despite the fact that ES have been used in tinnitus treatment since the1970s (studies testing different locations of the stimulating electrode and different parameters of the stimulating current) there are no recommendations on the adjustment of the stimulation conditions in tinnitus treatment. Two early studies on peripheral ES suggest that anodic (positive) ES, with an inhibitory potential should be used to suppress tinnitus; however, cathodic (negative) ES, bearing stimulating properties, should be used to excite the auditory nerve, evoking sound perception (Bochenek et al., 1989; Ren and Nuttall, 1995).

The aim of the study is to investigate the effect of applying positive and negative current polarities on the ear of a healthy subject and on the tinnitus ear of a patient with tinnitus. This comparison will further clarify the mechanisms of operation of non-invasive extra-cochlear ear ES. A second aim was to assess the effects of ES on tinnitus in tinnitus patients.

## Methods

The research was approved by Institutional Review Board of the Medical University of Lodz (RNN/251/05/KB) and was in accordance with the declaration of Helsinki. All patients gave their written, informed consent prior to inclusion in the study.

### Study sample

The research was conducted in the Department of Otolaryngology, Laryngological Oncology, Audiology and Phoniatrics, Medical University of Lodz. The material was composed of two groups (Table 1). Group I was a tinnitus group comprising 49 patients suffering from tinnitus (*n* = 71 tinnitus ears: 24 normal hearing ears and 47 sensorineural hearing loss ears). Twenty-eight of the participants were females and 21 were males, with an age range of 22–79 years (average = 53.4, *SD* = 15.6). In the case of unilateral tinnitus, only the tinnitus ear was tested. The allocation to tinnitus group was randomized, done according to the order of admission to our department. Group II was formed of healthy subjects. This group comprised 34 healthy, normal hearing individuals without tinnitus: 13 female and 21 male, mean age 23.5 years, range 20–35 years (*SD* = 2.9) (n–67 ears). The study was not blinded.

**Table 1:** Patient characteristics.

	**Tinnitus group (group I)**	**Healthy students group (group II)**
Number of participants	49 (22 patients with bilateral, 27-unilateral tinnitus)	34 (in 33 students both ears were tested, in 1 person - one ear was tested)
Number of tested ears	71	67
Gender	28 F, 21 M	13 F, 21 M
Age in years (mean ± standard deviation), range	53.4 ± 15.6 range: 22–79 year	23.5 ± 2.9 range: 20–35 year
Visual Analog Scale for tinnitus loudness (range 0–10) Mean ± standard deviation	Before ES: 5.52 ± 1.70 After ES: 3.27 ± 2.37	

Before the beginning of the study, otorhinolaryngological examination, and hearing tests (pure tone audiometry, speech audiometry, impedance audiometry, auditory brainstem responses) were conducted, as well as radiological diagnostics of head and cervical spine if necessary. Pathology in the external and/or the middle ear was an exclusion criterion, together with the presence of a pacemaker, CNS vascular malformation, epilepsy, or any history of head and neck neoplasm. Patients who reported tinnitus in the head but not in the ears were also excluded from the study. The patients from Group I were asked to assess tinnitus in visual analog scale (VAS) for loudness, directly before and directly after electrical stimulation. The scale ranged from 0 – no tinnitus, 1 – very quiet tinnitus to 10 – extremely loud tinnitus.

### Experimental procedure

ES was performed with the use of a custom-made apparatus supplied with four batteries of 1.5 V. The device allows for direct current stimulation within a frequency range of 0.25–8 kHz and an amplitude range of 0.01–2.24 mA. The external ear canal was filled with saline solution. The active silver probe was immersed inside external ear canal, avoiding contact with the skin of the canal. The passive electrode was placed on the forehead (in the midline) after skin abrasion with a suitable sterile abrasive electrode paste and clean gauze. The two electrodes were placed in such a way as to allow current transmission throughout the hypothetical plane (longitudinal axis) of the cochlea.

DC electrical stimulation was provided. The effects of positive current were first assessed. The series of tests with positive currents were completed then the same tests were carried out with negative currents. ES was performed using the following frequencies: 0.25, 1, 2, 3, 4, 5, 6, 7, and 8 kHz. For each stimulation frequency, the presence of an auditory precept (AP) was confirmed and, if AP was present, the minimum current amplitude necessary to produce AP was measured. ES treatment was started with a maximum well tolerated current intensity. If AP was present, the intensity of current was slowly decreased and the patient was asked to indicate the moment when the sound ceased to be audible. The protocol was performed first for positive (anodal), then for negative (cathodal) current in both groups, for each of the abovementioned frequencies.

The duration of the rectangular pulse depended on the frequency, e.g., for 250 Hz, one period lasted 4 ms (2 ms pulse and 2 ms pause). The voltage was constant and equaled 3 V. The maximum intensity was variable (ranged from 0.15 to 2.24 mA) and was applied according to the sensation of the patient. If the patient reported pain or other unpleasant sensation, the intensity of the current was smoothly decreased (with the intensity knob) to a level producing a tolerable sensation.

Next, the pitch of electrically-evoked AP was assessed in a sound proof chamber. A subgroup of 20 patients from the tinnitus group were selected. These perceptions were matched with free field sounds as pure tones from the audiometer. A pair of pure tones was delivered in a free field through the loudspeakers (Martin Audio C115), and the patient was asked to indicate the one which more closely resembled the sound perceived during ES. One tone was similar to the stimulating frequency and the other was next lower tone on the audiometer (Madsen Electronic Orbiter 922). If neither sound was similar, the next pair of tones was given. Matching was performed simultaneously with ES.

### Statistical testing

The numerical data was tested for normality of distribution using the Shapiro-Wilk *W*-test. Levene's test was performed in order to assess the homogeneity of variances in both compared groups. A p-level of 0.05 was assumed for all tests of significance. All the statistical procedures were conducted as two-tailed ones. Due to the small sample sizes and lack of normality, appropriate non-parametric tests were performed, and also robust standard errors or standard errors allowing for intragroup correlation were estimated when applicable.

Generalized estimating equations with clustered standard errors (i.e., allowing for intragroup correlation) were carried out for a numerical dependent variable (hearing threshold), a set of independent variables comprising current threshold values measured at selected frequencies, and subject hearing (hearing loss vs. normal hearing). All *p*-values obtained during the analysis approximated to a value of one.

## Results

Overall, 1,278 stimulations were conducted in the tinnitus group (71 ears × nine frequencies × two polarities), and 1,206 in the healthy student group (67 ears × nine frequencies × two polarities).

### The presence of AP during ES

In both groups, in the majority of cases, AP was produced during ES. Overall, it was present in 60 ears in the tinnitus group (84.5%) and in 66 ears in the healthy students group (98.5%). In the tinnitus group, from all the 1,278 stimulations, AP was evoked in 182 (14.24%) stimulations while no sound perception was evoked in the other 1,096 (85.76%). In the healthy students group, out of 1,206 stimulations, AP was evoked in 798 (66.2%) stimulations, while no AP was evoked in the other 408 (33.8%). The analysis showed that sound perception was less likely during ES in the tinnitus group than the healthy students group (*p* < 0.001). During ES temporary pain was reported by persons from both groups: in tinnitus group—by 25 patients (51%), and in healthy subjects group—by 14 individuals (41%). In all cases it was exclusively momentary and it disappeared as soon as current intensity was smoothly decreased, so the procedure was continued.

**Figure 1:**
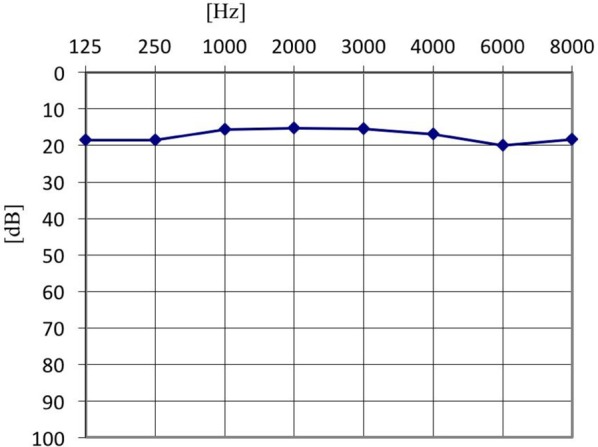
The averaged pure tone audiogram in tinnitus group–normal hearing tinnitus patients.

**Figure 2:**
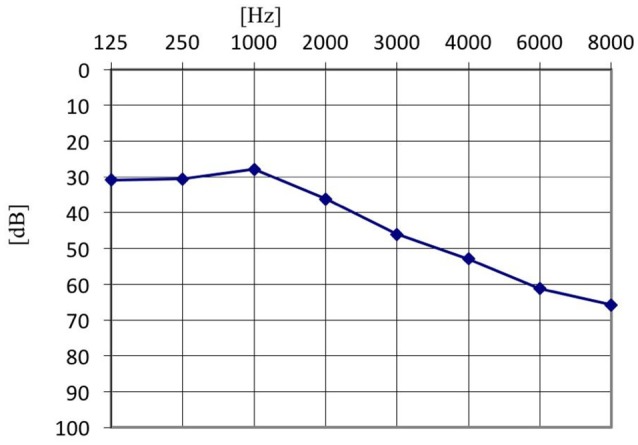
The averaged pure tone audiogram in the tinnitus group–hearing loss tinnitus patients.

**Figure 3:**
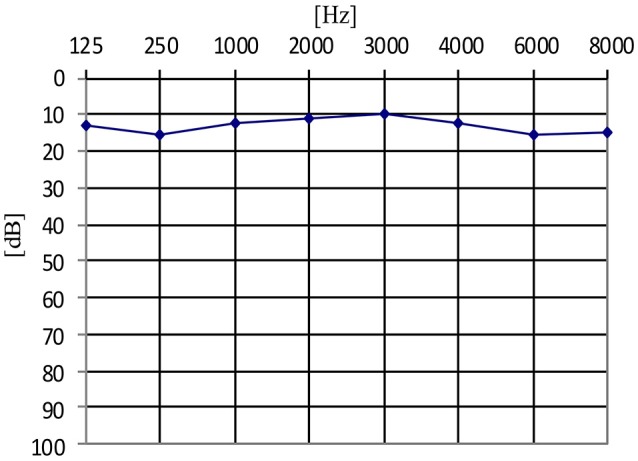
The averaged pure tone audiogram in the healthy students group.

Furthermore, in the tinnitus group we tested whether the presence of the AP depended on the type of tinnitus (tone vs. noise vs. tone + noise), age or hearing level. Binary logistic regression with robust standard error was performed (Table 2). No relationship was confirmed (OR = 0.42; 95% CI: 0.15–1.13; *p* = 0.085). AP was present in 91.1% of ears with tone-like tinnitus, in 70.0% in noise-like tinnitus and 80.0% of ears when tinnitus was a mixture of tone and noise. Within the tinnitus group, the presence of AP did not depend on age (OR = 0.97; 95% CI: 0.92–1.02; *p* = 0.172), nor hearing level (OR = 0.65; 95% CI: 0.09–4.63; *p* = 0.664). It was present in 71.4% of normal hearing ears, and in 71.6% of hearing loss ears. The mean age of patients with AP was 51.5 years (*SD* = 15.7), and the mean age of those without was 60.5 years (*SD* = 13.5). The averaged pure tone audiograms in the tinnitus group (normal hearing and hearing loss subjects) and in the healthy students group are presented in Figures 1–3.

**Table 2:** Logistic regression estimates for predictors of auditory perception (AP).

**Investigated trait**	**OR (95% CI)**	**Level of statistical significance (*p*-value)**
Type of tinnitus (tone vs. noise vs. tone + noise)	0.42 (0.15–1.13)	= 0.085
Age (1-year step)	0.97 (0.92–1.02)	= 0.172
Hearing level	0.65 (0.09–4.63)	= 0.664

### Analysis of current intensities needed to evoke AP

In the tinnitus group, the intensities of current evoking AP ranged from 0.16 to 1.67 mA (mean–0.614 mA), in the healthy students group—from 0.01 to 1.61 mA (mean–0.461 mA). The analysis showed that in the tinnitus group, ES needed higher intensities of current to evoke sound perception (*p* < 0.003). Moreover, in the tinnitus group we analyzed intensities of current evoking sound perception vs. averaged hearing thresholds at 0.25, 1, 2 kHz, respectively. We did not perform this analysis for higher stimulating frequencies since AP was practically absent for 4 kHz (present in five ears) and 8 kHz (present in three ears). There was no correlation between hearing threshold and current intensity needed to evoke AP during electrical stimulation.

### Analysis of current frequencies needed to evoke AP

**Figure 4:**
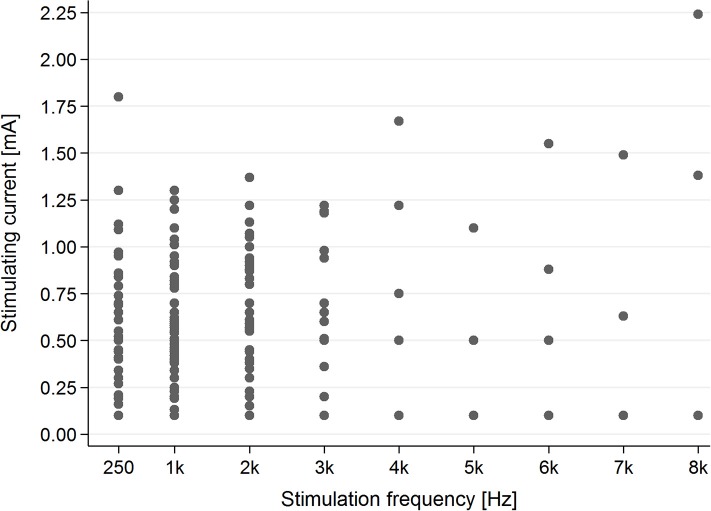
The presence of the AP in the tinnitus group for particular stimulating current parameters (mA/kHz).

In the tinnitus group, in the majority of cases (83.1%), AP was present for stimulating frequencies between 0.25 and 2 kHz (Figure 4).

**Figure 5:**
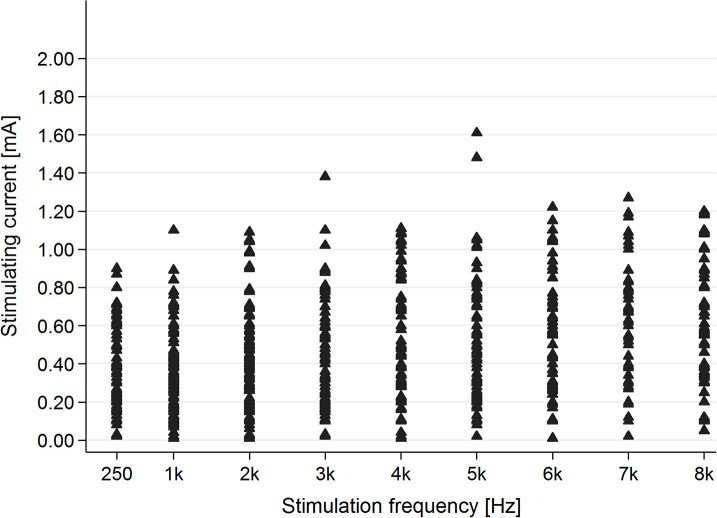
The presence of the AP in the healthy students group for particular stimulating current parameters (mA/kHz).

In the healthy students group, AP was present for each stimulating frequency—0.25–8 kHz (Figure 5).

### Correlation between frequency and intensity of current needed to evoke AP

**Figure 6:**
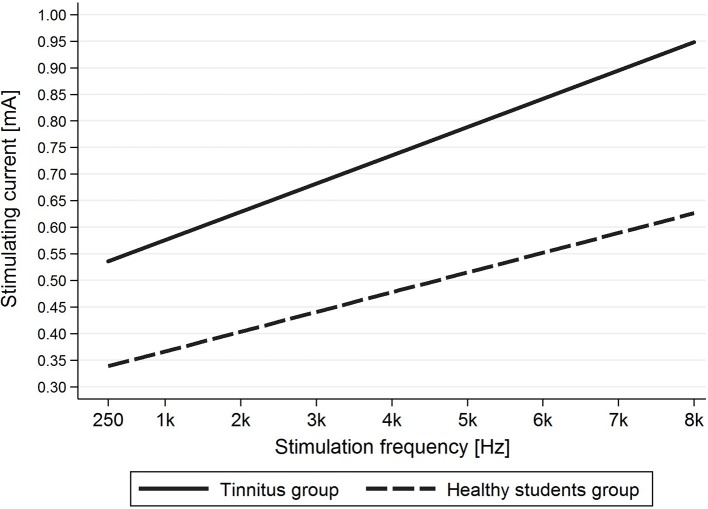
The dependence of stimulating current intensity on stimulating frequency.

Evaluation of the correlation between stimulating frequency and intensity of current indicated that higher stimulating frequencies needed higher current intensities to evoke AP in the tinnitus group (*p* < 0.001) as well as the healthy students group (*p* < 0.001) (Figure 6).

### Current polarity vs. AP vs. ear

**Figure 7:**
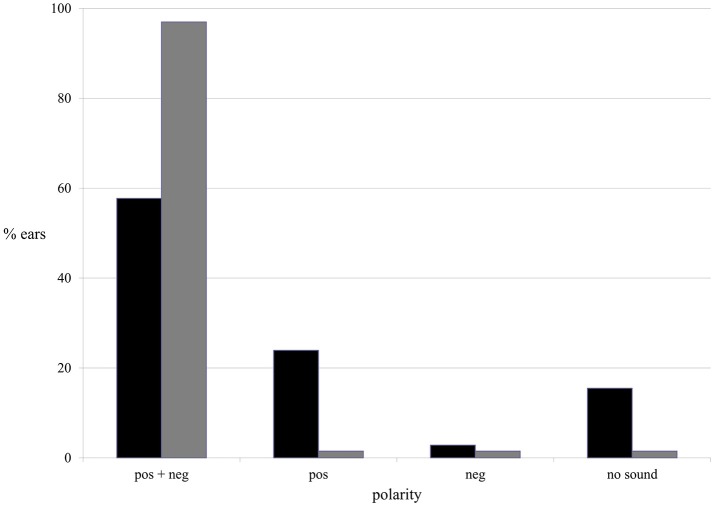
The presence of the AP in terms of current polarity in the tinnitus group (black column) and the healthy students group (gray column).

Both current polarities (positive and negative) evoked AP during ES. In the majority of ears AP was evoked by both positive and negative current: in 57.7% of tinnitus ears in the tinnitus group and in 97% of ears in the healthy students group (Figure 7).

The binary logistic regression with robust standard error was performed. Furthermore, in tinnitus group, with respect to AP, positive polarity appeared to be significantly more efficient than negative. AP was present in nearly 82% of tinnitus ears when positive current was used, and in 61% during negative stimulations (*p* < 0.001). In healthy students group, both polarities were equally effective, evoking AP in 98.5% of ears (*p* > 0.9).

In the tinnitus group, ES needed higher intensities of current to evoke AP for positive polarity and for the left ear compared with negative polarity and the right ear (Pos. left vs. Pos. right: *t* = 2.6041, *p* < 0.009; Pos. left vs. Neg. left: *t* = 2.511, *p* = 0.012). All other comparisons (*T*-tests) were not significant (*p* > 0.05). In the healthy students group, positive polarity needed higher intensities to evoke AP (*p* = 0.001), without significant differences between left and right ear in this group (*p* = 0.92).

### Matching electrically evoked AP with free field sounds in tinnitus group

**Figure 8:**
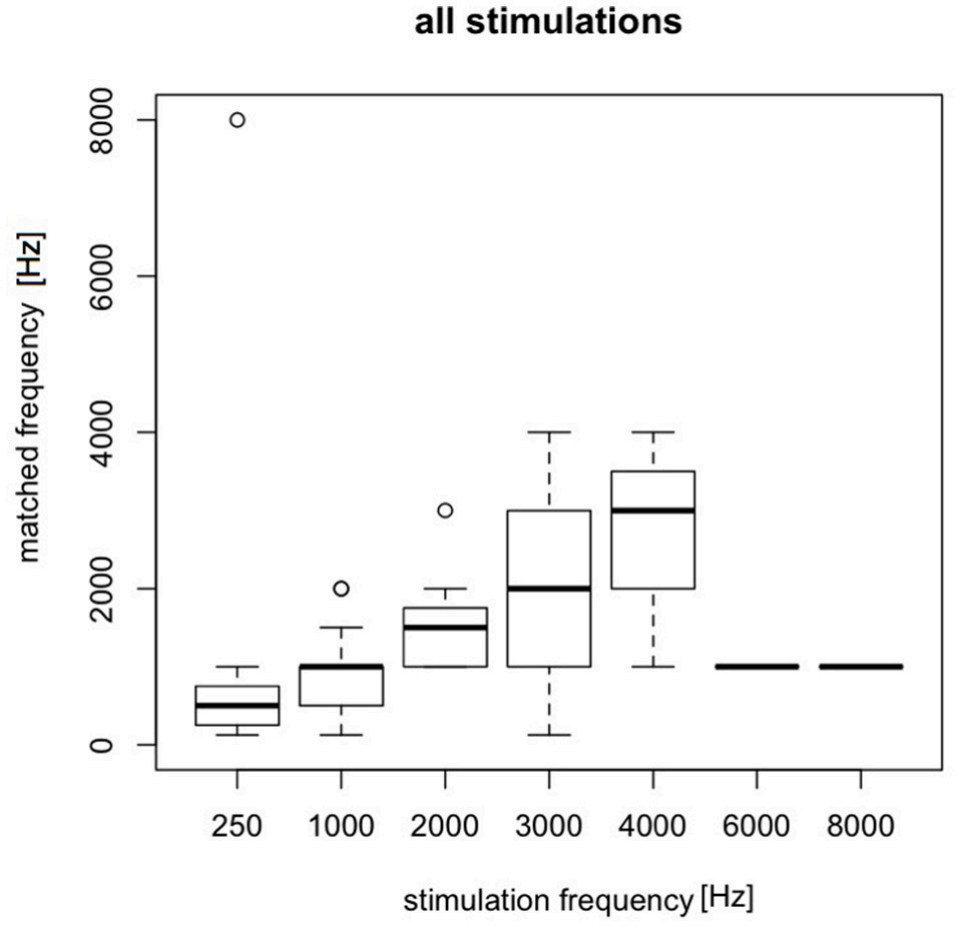
Matching electrically evoked pitch of AP with free field sounds in the tinnitus group.

The matching was done in tinnitus group in 20 patients. A total of 448 matchings were performed. Electrically-evoked AP were matched with free field sounds (pure tones from the audiometer). The pitch of electrically-evoked AP changed with a change in stimulating current frequency. Sound perceptions evoked by low stimulating frequencies (0.25–2 kHz) had more exact matching to free field sounds. 1 kHz appeared to have the most adequate matchings. 3 and 4 kHz were mainly identified as lower sounds like 2 and 3 kHz, respectively. 6 and 8 kHz were identified as low (1 kHz) sounds (Figure 8).

### Analysis of tinnitus after ES in tinnitus group

Generalized estimating equations with robust standard errors were carried out for a categorical dependent variable (an improvement in tinnitus) and a set of independent variables comprising sound perception and the subject status before and after stimulation.

**Figure 9:**
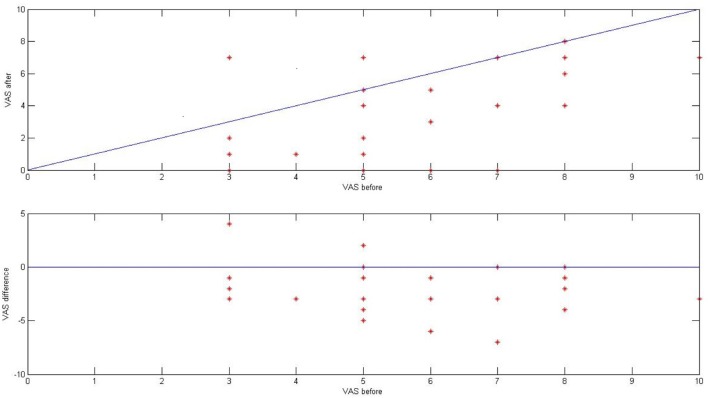
The effect of ES on tinnitus in the tinnitus group presented as a result in VAS for tinnitus loudness (before and after stimulation).

Before and directly after ES, we asked 28 patients from the tinnitus group (46 tinnitus ears) to describe the loudness of tinnitus in VAS (visual analog scale for tinnitus loudness). The mean tinnitus loudness before stimulation was 5.52 (*SD* 1.70), and after 3.27 (*SD* 2.37; *p* < 0.001) Figure 9.

Directly after ES, there was improvement in 21 ears (75%), no change in five ears (18%), and worsening in two ears (7%). In 10 out of 46 ears (22%), tinnitus disappeared. Interestingly, there was no correlation between the improvement in tinnitus and the presence of electrically evoked AP (*p* > 0.5).

## Discussion

In our research both positive and negative polarities excited the auditory system evoking an AP in the study participants. This effect, however, was more pronounced for positive polarity in no tinnitus and normal hearing individuals (healthy students group). In the tinnitus group the AP was present for a much narrower range of stimulating frequencies (0.25–2 kHz) when compared to the no tinnitus and normal hearing healthy students group (0.25–8 kHz).

In the tinnitus group, the current intensity needed to evoke an AP was higher than in the healthy students group, which could suggest the effect of hearing threshold. However, comparing normal hearing vs. hearing loss patients within tinnitus group, again, we did not observe any relationship between hearing threshold and current intensity evoking AP. Furthermore, we matched electrically evoked AP pitch with free field sounds in tinnitus group. We saw that the pitch of AP changed with a change in stimulating frequency. Afterwards, we analyzed the effect of multi-frequency ES on tinnitus. It appeared to be effective in 75% of tinnitus ears (with a high score of disappearance – 22%). The interesting fact was that the improvement in tinnitus was not correlated with the presence of AP during the ES.

Animal studies showed that electrical current influences the micromechanics of the Corti organ. Nuttall and Ren stimulating the round window membrane and cochlear duct in guinea pigs reported movements in the basilar membrane and emission of sound from the cochlea. This phenomenon was possible only when some OHCs were intact so their movements displaced the basilar membrane. The authors indicated that any intra- or extra-cochlear electric current stimulation affects the polarization of OHCs and could induce a traveling wave in the basilar membrane (Nuttall and Ren, 1995; Ren and Nuttall, 1995).

In our research, this mechanism of ES is also possible. Applying electric current to external ear canal we reach the cochlea (the conduction of the stimulus through the lining of the tympanic cavity and the round window) but since soft tissues have conductive properties, this might not be a single way in which electric impulse affects auditory system. Earlier research on ear ES demonstrated cortical potential alternations after ear ES, so we may alike influence central auditory system directly in the same time (Mielczarek et al., 2016). Furthermore, since the pitch of electrically evoked AP changed with a change of stimulating frequency, it is possible that we stimulate the cochlea (and auditory nerve) selectively, producing a normal traveling wave or directly changing (selectively) the OHC potential. However, the selectivity of the excitation is probably worst comparing to excitation of Corti organ with sound.

By applying negative pulses to the impaired cochlea, Portman et al. elicited auditory sensation, which was regarded as proof of auditory nerve excitation. This response, however, was not based on the stimulating frequency, as auditory sensation remained unchanged when the frequency was altered (Portmann et al., 1979).

Secondly, since in our research the AP was more pronounced in the group of healthy students (no tinnitus normal hearing young individuals) and since there were no differences between normal hearing and hearing loss patients in this regard within the tinnitus group, the authors assume that OHC function is not a determining factor for the AP phenomenon. We hypothesize that the condition of the synapsis and auditory neurons are responsible for these variations (Kujawa and Liberman, 2015; Viana et al., 2015).

Many studies describe the dependence of the ES effect on current polarity. Cazals et al. point to the direction of current flow applied to cochlea suggesting that positive polarity should be used to reduce tinnitus, and a negative polarity to evoke sound perception (Cazals et al., 1978). These findings have been confirmed elsewhere (Sun et al., 2000; Noreña et al., 2015), but the results seem to be inconsistent (Guo, 2001). Furthermore Cazals et al. claimed that when stimulating with negative current, the AP was present in a wider range of frequencies and lower intensities were required to produce auditory sensation. They placed the electrodes on the promontory or round window, and a reference one on the earlobe (Cazals et al., 1978). The results of our study showed that the direction of current flow (the polarity) is not a deciding factor whether ES evokes AP or not. In the healthy students group, AP appeared to be independent from the current polarity; however, in the tinnitus group, negative stimulations were less likely to evoke AP when compared to positive current. Negative stimulations needed lower intensities to evoke sound perception, which was coherent with Cazals et al.

AP evoked during stimulation of the deaf ears seems to be a confirmation that this perception is generated above the cochlea. Chouard et al. stimulating deaf ears (round window) registered auditory brainstem potentials and noticed sound sensation (AP) in almost all deaf ears (Chouard et al., 1979, 1994). These results may be considered proof for the concept that AP depends on auditory nerve function, not on the cochlea, although the cochlea acts as the first effector of the electric stimulus. Some authors evoke AP during ES on deaf ears (before cochlear implantation) to confirm restored function of the acoustic nerve (Gersuni and Volokhov, 1936; House and Brackmann, 1974; Bochenek et al., 1977; Portmann et al., 1979; Skarzynski et al., 1999). The use of promontory ES in patients labyrinthectomized due to Meniere's disease, resulted in sound percept despite complete loss of inner ear function (Lambert et al., 1990).

Rattay et al. suggest a correlation between polarity sensitivity and the state of degeneration and demyelination of peripheral neurons. According to them degenerated peripheral neurons would require lower anodic thresholds (Rattay et al., 2001). Our results do not confirm any such effect. In our research, in the tinnitus group, the intensity of current needed to evoke AP was higher than in healthy students group—suggesting the effect of neural degeneration due to natural processes in the tinnitus group, and the levels of current were higher for positive stimulation. Interestingly, in the tinnitus group, AP did not depend on hearing threshold. Since the two subgroups (tinnitus patients with normal hearing and tinnitus patients with hearing loss) were similar in terms of age, we can assume that the condition of the auditory nerve was similar, even if the hearing status differed. Viana et al. demonstrated in five “normal” ears, features of cochlear synaptopathy and the degeneration of cochlear nerve peripheral axons, in the presence of a near-normal hair cell population; they suggest that such changes account for human presbyacusis (Viana et al., 2015). Other animal studies showed that in age-related hearing loss, degeneration of cochlear synapses precedes both hair cell loss and hearing threshold elevation (Kujawa and Liberman, 2015).

In early research by Jellinek and Schreiber (1930) it was reported that ES of the ear with alternating current causes AP, which induces a sound perception at the stimulating frequency. The phenomenon was at first explained by vibratory or mechanical forces arising at the electrode, changes in the fluid volume (filling external ear canal) and mechanical excitation of the tympanum or ossicles. Afterwards Arapova et al. (1937) suggested another two possible mechanisms: the first was that AC evokes mechanical forces in the cochlea, thus stimulating the receptors in the usual way, while the second was that AC directly affects the receptor, omitting the stage of transformation from electrical to mechanical forces. For Gersuni and Volokhov, the proof of cochlea excitation was their production of beats by simultaneous electrical and sound stimulation at approximately similar frequencies (Gersuni and Volokhov, 1936).

Lusted and Simmons give two possible explanations for electrically-evoked AP: the OHC stimulation or afferent auditory fiber excitation (Lusted et al., 1984). Stevens et al. accounts for the “electrophonic effect” by direct stimulation of the auditory nerve, and by electromechanical transduction in the inner ear or tympanum (Stevens and Jones, 1939). Kellaway argues in favor of cochlear structure excitation: sound sensation was always a pure tone, never noise, the change in the polarity of current did not change the character of the sound effect, the absence of tympanic membrane did not influence the perceived sound (Kellaway, 1946). In our research, the AP was a pure tone sound, never noise, suggesting the involvement of sensory elements rather than mechanical. Furthermore, it did not depend on current polarity, since it was present for both.

Many papers have confirmed the value of ES of the ear in tinnitus treatment (Cazals et al., 1978; Portmann et al., 1979; Rubinstein et al., 2003; Arts et al., 2015). Our study proved that extracochlear ES with positive and negative current was efficient to stimulate the auditory system. The auditory nerve appeared to be the most probable place of AP generation. Stimulating tinnitus ears with two polarities, we obtained a higher ratio of improvement (75%) comparing to positive stimulation alone (Mielczarek and Olszewski, 2014). However, since the presence of AP was not correlated with the improvement in tinnitus, some other mechanisms may account for this effect.

## Author contributions

MM, AN, WS, and JO: Substantial contribution to the design of the study and the draft of the manuscript; MM and JO: Data acquisition; WS and MM: Performed statistical testing; MM, WS, and AN: Created figures. All authors revised and approved the manuscript.

### Conflict of interest statement

The authors declare that the research was conducted in the absence of any commercial or financial relationships that could be construed as a potential conflict of interest.
